# Randomized prospective trial comparing ejaculatory preservation HoLEP versus standard HoLEP: the other face of the coin

**DOI:** 10.1007/s00345-024-05418-y

**Published:** 2025-03-03

**Authors:** Ahmed Eliwa, Ali Aldarraji, Khaled Abdelwahab, Emad Salem

**Affiliations:** Department of Urology, Zagazig University Hospitals, Zagazig, Egypt

**Keywords:** EP HoLEP, BPH, Holmium, Laser, Benign prostate hyperplasia, Ejaculation preservation, Urinary incontinence, Continence

## Abstract

**Background:**

Laser Anatomical Endoscopic Enucleation of the Prostate (LAEEP) techniques offer a promising solution for patients seeking to alleviate urinary symptoms while preserving sexual function. The ejaculation preservation approach has been shown to achieve an impressive 90% success rate in maintaining antegrade ejaculation.

**Purpose:**

To explore the effect of the ejaculatory preservation HoLEP technique on postoperative continence and ejaculation.

**Patients and methods:**

We conducted a prospective randomized study adhering to CONSORT guidelines. Patients with prostate sizes between 40 and 80 gm, IPSS scores > 20, Qmax < 10 ml/s, and IIEF-5 scores  > 22 were eligible for inclusion. A total of 43 patients were randomized into two groups: Group 1 (n = 22) underwent HoLEP, while Group 2 (n = 21) underwent EP HoLEP. We assessed functional outcomes and ejaculatory dysfunction at baseline and 1-, 3-, and 6- month. The study period was from October 2022 to March 2024.

**Results:**

Data from 40 patients were analyzed. Functional outcomes showed significant improvement in Group 1, with IPSS (median 12.5, P < 0.006) and Qmax (median 15, P < 0.04) at 3-month. ICIQ-UI SF scores showed a significant difference in the incontinence episodes (domain Q3), which were lower (P < 0.023) in Group 2 compared to Group 1 during the 1-month visit. MSHQ-EjD-SF scores were significantly higher in Group 2 at both 3- and 6- month (P < 0.01 and P < 0.02, respectively). IIEF-5 scores showed no significant difference between both groups during the study period.

**Conclusion:**

Implementing the ejaculation preservation technique during HoLEP appears to improve early postoperative continence and preserve ejaculation.

**Supplementary Information:**

The online version contains supplementary material available at 10.1007/s00345-024-05418-y.

## Introduction

The groundbreaking research conducted by Peter Gilling in 1998, introducing Holmium Laser Enucleation of the Prostate (HoLEP), has revolutionized the treatment landscape for benign prostatic hyperplasia (BPH) [1]. This innovation paved the way for the widespread adoption of Laser Anatomical Endoscopic Enucleation of the Prostate (LAEEP) techniques. Subsequently, novel surgical methods, such as direct bladder neck incision, early apical release, and 3-horse shoe-like incision, have been implemented to further enhance outcomes and minimize perioperative complications associated with standard HoLEP [2–5]. Despite these advancements, HoLEP is not without its challenges, as complications like transient urinary incontinence (UI) and retrograde ejaculation (RE) remain prevalent. Studies have reported that UI affects 3.3% to 26% of patients, with most regaining continence within a year, while RE impacts approximately 64.7% of patients following HoLEP [6, 7].

Numerous clinical studies emphasize the crucial role of the musculus ejaculatorius (supracollicular and paracollicular) in preserving ejaculation [8–10]. On the other hand, there is an ongoing debate regarding the bladder neck sphincter's contribution to maintaining ejaculation and continence [11]. Our objective is to investigate the hybrid effects of the Ejaculatory Preservation (EP) HoLEP technique compared to standard HoLEP on early postoperative continence and antegrade ejaculation.

## Patient and methods

### Study design

This prospective randomized single-center study was conducted in adherence to the CONSORT guidelines.

### Study population

Eligible patients who met the inclusion criteria and scheduled for surgical treatment due to benign prostatic obstruction (BPO) caused by benign prostatic hyperplasia (BPH). The study was approved by the ethical committee (ZU-IRB No. 9938). Written informed consent was obtained from all patients, and the study period was from October 2022 to March 2024.

### Inclusion criteria

Patients with BPH and prostate size between 40 and 80 g as measured by transrectal ultrasound (TRUS), An International Prostate Symptom Score (IPSS) greater than 20, A maximum urinary flow rate (Qmax) of less than 10 ml/s. Sexually active individuals with a normal marital life with International Index of Erectile Function-5 (IIEF-5) score more than 22.

### Exclusion criteria

Patients with systemic disease, diabetes mellitus, or neurological disorder having an impact on antegrade ejaculation. Patients with bladder or urethral pathology.

### Sample size

Assuming an overall satisfaction score of 6 ± 2.4 for the Ejaculatory Hood Spring compared to 4 ± 2.1 for the standard group [12], with a power of 80% and a 95% confidence interval, and accounting for a 10% dropout rate during follow-up, the estimated sample size was 40 cases, with 20 cases in each group. (Open Epi, Version 3.01). This study was designed to detect the rate of retrograde ejaculation (RE) as the primary outcome and evaluate the urinary incontinence (UI) rate as the secondary outcome.

### Randomization

Patients were randomized using a computer-generated allocation sequence. A total of 48 patients were assessed for eligibility, and ultimately, 43 patients were randomly assigned in a 1:1 ratio to receive HoLEP or EP HoLEP.

### Outcome measures

The primary endpoints of the study included early postoperative continence assessment at 1- and 3-month, measured by the International Consultation on Incontinence Questionnaire-Urinary Incontinence Short Form (ICIQ-UI SF) in both groups. Secondary endpoints included changes in Male Sexual Health Questionnaire-Ejaculatory Dysfunction Short Form (MSHQ-EjD-SF) scores from baseline to postoperative, IIEF-5, and the assessment of lower urinary tract symptoms (LUTS) relief using IPSS, Qmax, and quality of life (QoL). Perioperative adverse events were also compared and reported. We defined postoperative UI as reported by a patient (stress UI, urge UI, and dribbling after urination).

### Perioperative workup

The baseline workup included a clinical examination, digital rectal examination. Laboratory and radiological evaluations consisted of serum prostate-specific antigen (PSA), pelvic-abdominal ultrasound, prostate volume measurement by (TRUS), and uroflowmetry. All patients were assessed using the IPSS, Qmax, QoL, IIEF-5, and MSHQ-EjD-SF both preoperatively and postoperatively.

### Description of equipment and surgical technique

A 26F Storz continuous flow resectoscope with separate laser bridge and 0.9% saline was utilized. The laser equipment used included 550 μm fiber and a 100W holmium-YAG machine (Sphinx® LISA Laser Machine, Germany), set to a power of 1.5–2 J at 40–50 Hz. Resection of the enucleated prostatic adenoma was performed using a Storz standard bipolar cutting loop, followed by retrieval of residual tissue with an Ellik evacuator. At the end of the surgery, a 22-F three-way catheter was inserted and retained until the urine was clear.

### Surgical technique

**Fig. 1:**
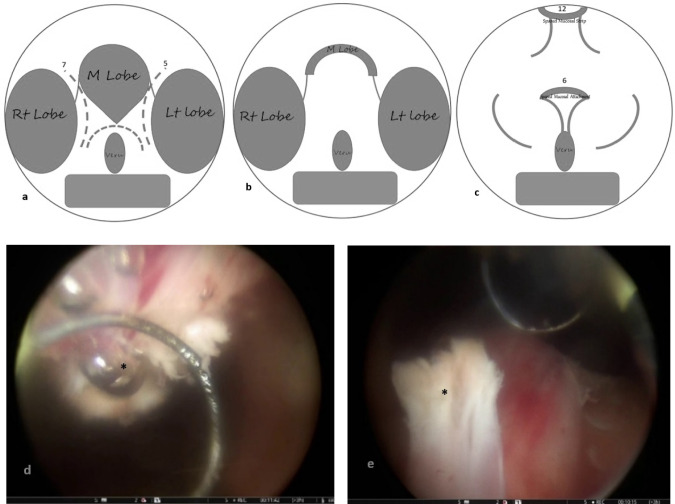
Illustrates the ejaculation preservation technique. **a** Identify the prostate's anatomical region (Tri/Bilobar) and create incisions at 5 and 7 o'clock, joining them 10 mm above the veru. **b** Completely dissect the middle lobe, leaving it attached at the bladder neck. **c** Preserve the mucosal strip at 2 and 10 o’clock, extending from the bladder neck (12 o'clock) till the level of the veru. **d** Spare the mucosal strip at 2 and 10 o'clock, connecting at 12 o'clock, Strike*. **e** Preserve mucosal attachments to the bladder neck from the middle lobe at 5 and 7 o'clock, Strike*

Two experienced surgeons performed the procedures, each having completed over 180 HoLEP surgeries. All patients received prophylactic intravenous antibiotics 60 min prior to surgery, which was conducted under spinal anesthesia. In Group 1, the standard HoLEP enucleation was performed as outlined by Gilling [1]. In Group 2, we conducted Ejaculatory Preservation (EP) HoLEP by implementing a preservation maneuver during the trilobar gland enucleation of the median lobe. This involved leaving approximately 10mm proximal to the verumontanum and 5mm paracollicular of the lateral lobes intact (Fig. 1a). Additionally, 5mm of mucosal attachments to the bladder neck were preserved by trimming the bladder neck at the 5 and 7 o’clock positions (Fig. 1b). At the 2 o’clock and 10 o’clock positions, we spared the mucosal strip extending from the bladder neck at the 12 o’clock position down to the urethral lumen at the level of the verumontanum (Fig. 1c). For the bilobar gland, the incision was made at the bladder neck at the 5 and 7 o’clock positions and extended distally along the capsular plane to 10mm proximal to the verumontanum. An intact mucosal strip was maintained from the bladder neck at the 6 o’clock position to the verumontanum. The lateral lobes were enucleated using the same technique applied to the trilobar gland.

### Postoperative follow-up

Patients were scheduled for follow-up at 1-, 3-, and 6-month post-surgery. Outcome measured were ICIQ-UI SF, IPSS, Qmax, QoL, IIEF-5, and MSHQ-EjD-SF. Delayed complications were addressed. Patients were also advised to resume sexual activity after catheter removal.

### Statistical analysis

Statistical analysis was conducted using SPSS V23.0 (IBM Corp, Armonk, NY). Parametric data were analyzed with an unpaired Student’s t-test, categorical data with the Chi-square or Fisher's exact test, and nonparametric data, presented as median and range, with the Mann–Whitney test. The significance level was set at P < 0.05.

## Results

A total of 48 patients met the eligibility criteria, with 43 patients randomly assigned to two groups: (n = 22; Group 1) underwent HoLEP, and (n = 21; Group 2) underwent EP HoLEP, study flow chart is provided in Supplementary Materials (Fig. S1). The baseline characteristics, perioperative data and complications were comparable in both groups, as shown in Table 1. Both groups demonstrated statistical improvements in functional urinary outcomes, as measured by IPSS, Qmax, and QoL, compared to baseline. However, at 3-month, Group 1 exhibited significant difference in IPSS (median 12.5, P < 0.006) and Qmax (median 15, P < 0.04), with no significant differences found between both groups at the 6-month follow-up, as shown in Table 2.

**Table 1:** Baseline characteristics, perioperative data, and complications of patients

Baseline characteristics	Group 1 (n = 20; HoLEP)	Group 2 (n = 20; EP HoLEP)	P
Age (years), mean ± SD	63.9 ± 2.5	65.2 ± 3.7	0.73
BMI (kg/m2), mean ± SD	28.2 ± 3.15	27.8 ± 3.17	0.69
Prostate volume (ml), mean ± SD	57.6 ± 9.8	54.1 ± 9.3	0.26
PSA (ng/ml), mean ± SD	1.63 ± 0.7	1.77 ± 0.85	0.56
IPSS, median (range)	25 (18–32)	25 (20–32)	0.67
QoL, median (range)	4 (3–5)	5 (3–5)	0.58
Qmax (ml/s), median (range)	6 (4–9)	6.5 (4–10)	0.53
IIEF-5s, median (range)	22 (19–25)	22 (19–25)	0.59
Perioperative data and complications			
Enucleation Time (min), mean ± SD	35.4 ± 5.6	35.6 ± 6.5	0.89
Intraoperative complication None, n (%) Capsular injury, n (%) Capsular perforation, n (%)	18 (90%) 1 (5%) 1 (5%)	18 (90%) 2 (10%) 0 (0%)	0.51
Catheter time, mean ± SD	5.6 ± 1.69	5.2 ± 1.59	0.5

The changes in urinary and sexual function during the preoperative and postoperative periods									
	Preoperative		P	3 months postoperative		P	6 months postoperative		P
	Group 1 (n = 20; HoLEP)	Group 2 (n = 20; EP HoLEP)		Group 1 (n = 20; HoLEP)	Group 2 (n = 20; EP HoLEP)		Group 1 (n = 20; HoLEP)	Group 2 (n = 20; EP HoLEP)	
IPSS, median (range)	25 (18–32)	25 (20–32)	0.67	12.5 (9–19)	11 (7–17)	0.006*	9 (4–19)	8 (5–27)	0.41
Qmax (ml/s), median (range)	6 (4–9)	6.5 (4–10)	0.53	15 (13–22)	14 (11–18)	0.04*	17 (13–21)	16 (9–19)	0.96
QoL, median (range)	4 (3–5)	5 (3–5)	0.58	2 (1–4)	2 (1–4)	0.84	3 (1–5)	2 (1–4)	0.61
IIEF-5s, median (range)	22 (19–25)	22 (19–25)	0.59	22 (20–25)	22 (19–25)	0.8	22 (17–25)	22 (17–25)	0.77

*P < *0.05*

**Table 2:** The changes in ICIQ-UI SF and MSHQ-EjD-SF domains

	Group 1 (n = 20; HoLEP)	Group 2 (n = 20; EP HoLEP)		Group 1 (n = 20; HoLEP)	Group 2 (n = 20; EP HoLEP)	
Postoperative ICIQ-UI SF						
	1 month postoperative		P	3 months postoperative		P
Q3, median (range)	3.5 (0–5)	1 (0–4)	0.023*	0 (0–3)	0 (0–4)	0.98
Q4, median (range)	2 (0–4)	0 (0–2)	0.23	0 (0–4)	0 (0–2)	0.92
Q5, median (range)	3 (0–6)	0 (0–7)	0.58	0 (0–4)	0 (0–6)	0.84
Total	5 (0–13)	1 (0–11)	0.61	0 (0–9)	0 (0–12)	0.79

Postoperative MSHQ-EjD-SF						
	3 months postoperative		P	6 months postoperative		P
Q1, median (range)	1 (1–5)	5 (1–5)	0.004*	1 (1–5)	5 (1–5)	0.018
Q2, median (range)	0 (0–5)	5 (0–5)	0.011*	0 (0–5)	5 (0–5)	0.03*
Q3, median (range)	1 (0–5)	5 (0–5)	0.012*	0 (0–5)	5 (0–5)	0.03*
Q4, median (range)	4 (0–5)	0 (0–5)	0.02*	5 (0–5)	0 (0–5)	0.01*
Total	6.5 (1–15)	15 (6–15)	0.01*	6 (1–15)	15 (6–15)	0.02*

*P < 0.05

**Fig. 2:**
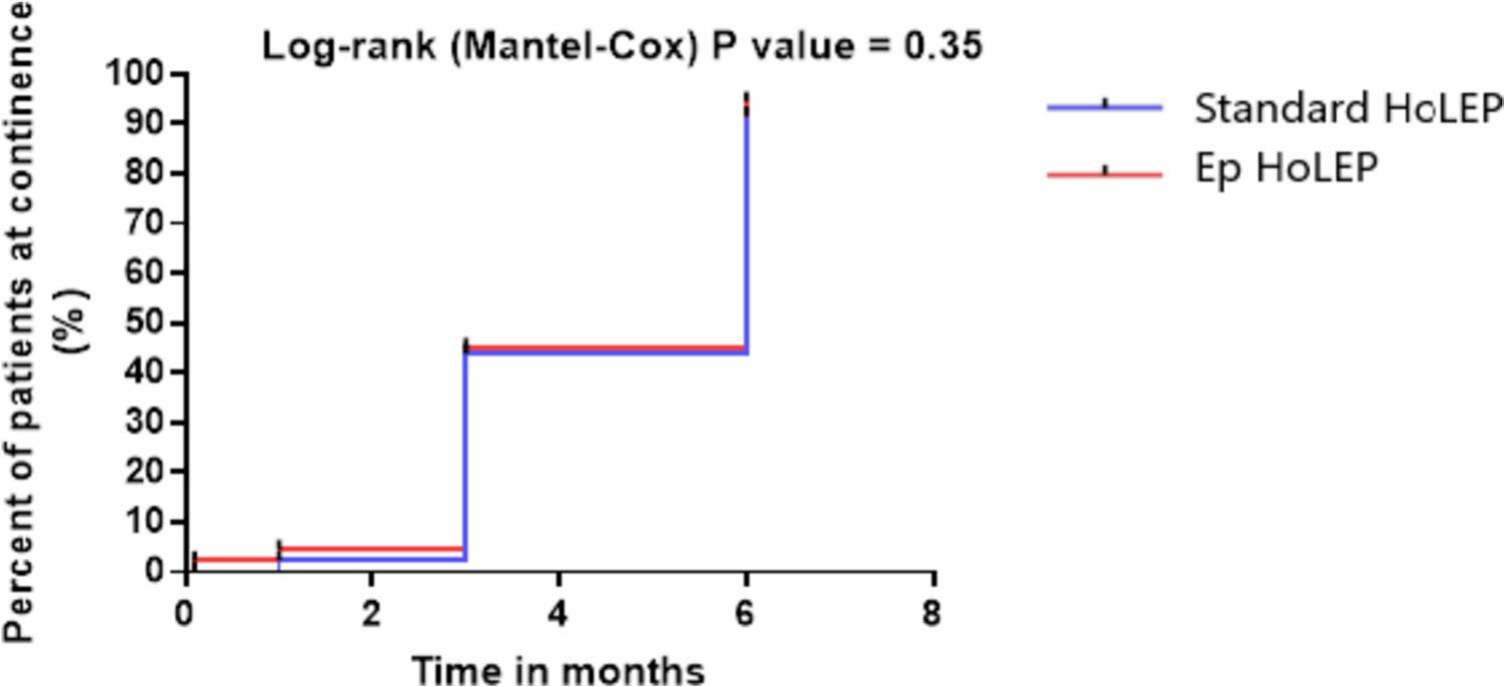
Kaplan–Meier curve showing the proportion of patients with continence at 3- and 6- month post-intervention

ICIQ-UI SF scores showed no significant difference between the two groups. However, Group 2 demonstrated a significant reduction in the frequency of UI episodes related to urine leakage (domain Q3) compared to Group 1 during the 1-month visit (P < 0.023), as shown in Table 2. The erectile function assessment (IIEF-5) results were comparable between the groups, as presented in Table 2. Group 2 achieved higher overall MSHQ-EjD-SF scores at the 3- and 6-month follow-ups (P < 0.01 and P < 0.02, respectively), which observed in domains Q1, Q2, and Q3. Conversely, domain Q4 indicated that lower scores were associated with greater satisfaction. In contrast, Group 1 exhibited declining scores in domains Q1, Q2, and Q3, which correlated with reduced satisfaction in domain Q4, as detailed in Table 2. Kaplan–Meier curves showed earlier recovery of continence rates in Group 2 compared to Group 1 within the first 3- month. However, no significant differences were observed between the groups thereafter (Fig. 2).

## Dissuasion

The HoLEP procedure has been shown to achieve satisfactory symptomatic and functional outcomes in patients with bladder outlet obstruction (BOO) [13]. However, retrograde ejaculation and postoperative urinary incontinence remain major concerns. Urologists performing HoLEP face the challenge of alleviating LUTS while preserving ejaculatory function in sexually active men.

In our study, we explore the effect of the ejaculatory preservation technique on achieving urinary continence while ensuring antegrade ejaculation. This personalized technique demonstrated a significant improvement in IPSS scores at 3-month, with lower scores in Group 2 compared to Group 1 (median 11 vs. 12.5, P < 0.006). However, by 6-month no significant difference was observed (median 9 vs. 8, P > 0.41). These findings align with Kim et al., who reported IPSS improvements at 3 months for conventional HoLEP and EH-HoLEP (8.2 and 7.3, respectively) with no significant difference between the groups [14]. Similarly, Rouf et al.’s reported a mean IPSS scores of 3.92 for the ep HoLEP group at 6-month [13]. Variations in IPSS values across studies may be attributed to differences in higher preoperative scores.

Our study demonstrated improved QoL scores in both groups, consistent with findings by Kim et al., who reported similar QoL scores at 3-month [14]. In contrast, Xu et al. observed better QoL scores in the modified HoLEP group compared to the traditional group, attributing this to the role of RE on QoL scores [15].

Our results showed a significant increase in Qmax at the 3-month follow-up in Group 1 compared to Group 2 (15 vs. 14, P < 0.04), possibly due to the more preservative technique used in Group 2. However, this difference was not observed at the 6-month follow-up. This aligns with findings by Xu et al., who reported a significantly lower Qmax in the modified HoLEP group at 1-month compared to the traditional group (P < 0.005), with no subsequent significant differences [15]. Kim et al. found mean Qmax of 21.2 for EH-HoLEP and 18.1 for conventional HoLEP at 3-month, with no significant differences between the groups [14].

At the 1-month visit, the response to Q3 of the ICIQ-UI SF (“How often do you leak urine?”) demonstrated a significantly lower UI rate in Group 2 compared to Group 1 (P < 0.023). Our ejaculatory preservation HoLEP technique spares the apical tissue, as well as the dorsal and ventral mucosal strips extending from the bladder neck to the verumontanum, which may explain the early continence recovery. Similarly Xu et al. while preserving mucous membrane of the bladder neck between the 11 and 1 o’clock positions had a low incidence (1.03%) of UI and RE (33.3%) [15]. Additionally, Press et al. when selectively enucleated large median lobes, reported no instances of stress UI and high rates of normal ejaculation [16]. However, it is important to acknowledge differences in surgical techniques, outcome measures (e.g., definitions of post-enucleation UI), and study durations, which may account for variability in the reported outcomes.

Our study found no significant differences in IIEF-5 scores between the two groups over study period.

These findings align with Long et al. and Kim et al., who also reported no significant differences in IIEF-5 scores between their groups [14, 17]. In contrast, Rouf et al. reported lower IIEF-5 scores in the EP-HoLEP group at both 3- and 6-month [13]. This discrepancy may be attributed to their use of question 5 of the IIEF-5 questionnaire to assess overall sexual satisfaction.

In the current study, Group 2 demonstrated significantly higher MSHQ-EjD-SF scores at both 3- and 6-month follow-ups (P < 0.01 and P < 0.02, respectively). In contrast, Rouf et al. reported a deterioration in ejaculation scores at 3- and 6- month following EP-HoLEP, attributing this to the inclusion of larger prostate volumes compared to those in our study [13]. Kim et al., when evaluating HoLEP with ejaculatory hood-sparing, found that their technique did not improve antegrade ejaculation, highlighting the necessity of apical tissue preservation to maintain antegrade ejaculation [14]. Long et al. reported a low rate of RE with their technique, although their study did not specifically address urinary incontinence [17].

The literature shows a wide diversity in ejaculation preservation techniques, energy sources, and heterogeneity in the tools used to measure outcomes. The strength of the current study lies in comparing ejaculation technique with standard technique for urinary continence in a randomized trial. The limitation of our study is the small sample size, with the sample calculation based on retrograde ejaculation as the primary outcome, which may introduce potential selection bias. However, the urinary incontinence rate was thoroughly evaluated as a secondary outcome using both objective and subjective scales. Further studies are needed to assess the generalizability of our conclusion.

## Conclusion

Implementing the ejaculation preservation technique during HoLEP appears to improve early postoperative continence and preserves ejaculation.

## Supplementary Information

Below is the link to the electronic supplementary material.

Supplementary file1 (DOCX 21 KB)

Supplementary file2 (mp4 115984 KB)

## Data Availability

The datasets generated and analyzed during the current study are available from the corresponding author upon reasonable request.

## Abbreviations

BPH: Benign prostatic hyperplasia
BPO: Benign prostatic obstruction
EP: Ejaculatory preservation
LAEEP: Laser anatomical endoscopic enucleation of the prostate
HoLEP: Holmium laser enucleation of the prostate
EP HoLEP: Ejaculatory preservation Holmium laser enucleation of the prostate
LUTS: Lower urinary tract symptoms
IPSS: International Prostate Symptom Score
TRUS: Transrectal ultrasound
QoL: Quality of life
Qmax: Maximum urinary flow rate
IIEF-5: International Index of Erectile Function-5
ICIQ-UI SF: International Consultation on Incontinence Questionnaire-Urinary Incontinence Short Form
MSHQ-EjD-SF: Male Sexual Health Questionnaire-Ejaculatory Dysfunction Short Form
PSA: Prostate-specific antigen
RE: Retrograde ejaculation
SUI: Stress urinary incontinence
UI: Urinary incontinence
UUI: Urgent urinary incontinence
BOO: Bladder outlet obstruction
