# Supplementary material for: Randomized prospective trial comparing ejaculatory preservation HoLEP versus standard HoLEP: the other face of the coin
Source: World J Urol. 2025 Mar 3;43(1):145. doi: 10.1007/s00345-024-05418-y (PMC11876214; doi:10.1007/s00345-024-05418-y)
Supplement: Supplementary file 1 — Supplementary file1 (DOCX 21 KB) [file 345_2024_5418_MOESM1_ESM.docx]

**Fig. S1. Study flowchart.**
